# Calcium Chloride vs. Mechanical Preparation of Fibrinogen-Depleted Human Platelet Lysate: Implications for Umbilical Cord Mesenchymal Stem Cell Culture

**DOI:** 10.3390/life15010012

**Published:** 2024-12-27

**Authors:** Yen Theng Lim, Muttiah Barathan, Yu Ling Tan, Yi Ting Lee, Jia Xian Law

**Affiliations:** Department of Tissue Engineering and Regenerative Medicine, Faculty of Medicine, Universiti Kebangsaan Malaysia, Cheras, Kuala Lumpur 56000, Malaysia; a189080@siswa.ukm.edu.my (Y.T.L.); barathanmuttiah@ukm.edu.my (M.B.); tan970812@gmail.com (Y.L.T.); yitingyyl428@gmail.com (Y.T.L.)

**Keywords:** fibrinogen, human platelet lysate, mesenchymal stem cell, growth factor, cell proliferation

## Abstract

Fetal bovine serum (FBS) has long been the standard supplement in cell culture media, providing essential growth factors and proteins that support cell growth and differentiation. However, ethical concerns and rising costs associated with FBS have driven researchers to explore alternatives, particularly human platelet lysate (HPL). Among these alternatives, fibrinogen-depleted HPL (FD-HPL) has gained attention due to its reduced thrombogenicity, which minimizes the risk of clot formation in cell cultures and enhances the safety of therapeutic applications. This study investigates two preparation methods for FD-HPL from human platelet concentrates: the calcium chloride method and a mechanical approach. The concentrations of critical growth factors, including vascular endothelial growth factor (VEGF), brain-derived neurotrophic factor (BDNF), insulin-like growth factor (IGF), and keratinocyte growth factor (KGF), were evaluated for both methods. Additionally, the impact of FD-HPL on the proliferation and morphology of umbilical cord-derived mesenchymal stem cells (UC-MSCs) was assessed. The findings revealed that the calcium chloride method produced significantly higher concentrations of all measured growth factors compared to the mechanical method. Moreover, UC-MSCs cultured in calcium chloride-prepared FD-HPL exhibited enhanced cellular characteristics, including increased cell size, elongation, and improved overall morphology compared to those cultured in mechanically processed FD-HPL. These results indicate that the preparation method significantly influences the biological properties of HPL and the effectiveness of UC-MSC culture. The calcium chloride method emerges as a superior technique for producing FD-HPL, offering a promising alternative to FBS in regenerative medicine applications. This study underscores the importance of preparation methods in optimizing HPL for cell culture and therapeutic uses.

## 1. Introduction

Fetal bovine serum (FBS) has long been a crucial component in cell culture media due to its rich supply of growth factors, hormones, and other proteins [1]. These include pregnancy hormones such as estrogen, progesterone, and prolactin, as well as growth factors like epidermal growth factor (EGF), fibroblast growth factor (FGF), and platelet-derived growth factor (PDGF). Additionally, FBS contains attachment proteins such as fibronectin, collagen, and laminin, which facilitate cell adhesion to the culture substrate, along with macromolecules like albumin, transferrin, and immunoglobulins that provide essential nutrients and help remove cellular waste products [2]. Despite the benefits of FBS, its limited availability and rising costs have spurred research into serum-free and xeno-free culture systems, particularly for specific cell types such as endothelial progenitor cells [3]. However, transitioning to FBS-free media poses challenges, and further research is needed to develop formulations that can fully replace FBS without compromising cell viability or function [4].

The search for viable alternatives reflects the growing emphasis on ethical and practical improvements in biomedical research. Alternatives to FBS, such as human serum, have demonstrated similar efficacy in supporting stem cell proliferation and differentiation, potentially mitigating the health risks associated with FBS [5]. Other serum types, such as bovine and newborn calf serum, have also shown varying success in promoting cell growth [6]. Additionally, human platelet lysate (HPL) has emerged as a promising alternative to FBS, particularly for human cell culture. HPL is rich in growth factors like PDGF, transforming growth factor beta (TGF-β), and EGF, which promote cell proliferation and differentiation, making it highly effective for expanding stem cells, especially mesenchymal stem cells (MSCs) [7]. Unlike FBS, HPL is derived from human sources, reducing the risk of xenogeneic immune reactions and improving batch-to-batch consistency [8]. Furthermore, HPL avoids the ethical concerns associated with animal-derived products, making it more suitable for clinical applications in regenerative medicine [9]. A systematic review has shown that HPL can support similar or even enhanced cell growth compared to FBS, providing a viable xenogeneic-free option for biomedical research and therapeutic use [10].

However, challenges remain in the use of HPL, particularly fibrinogen-induced clotting, which can disrupt cell culture environments. Clot formation can interfere with media preparation, storage, and cell culture processes, leading to inconsistencies in experimental conditions and outcomes [11]. Fibrinogen-depleted HPL (FD-HPL) has been developed to address this issue, supporting MSC expansion without the need for heparin, while maintaining viability, proliferation, and immunomodulatory properties [12]. Various methods can effectively remove fibrinogen from HPL, including calcium-induced clotting, mechanical hydrogel disruption, centrifugation, sonication, freeze–thaw cycles, and calcium activation [13,14]. The freeze–thaw method is commonly used for platelet lysis and growth factor release, while sonication requires specialized equipment. Calcium activation induces platelet degranulation, and mechanical disruption physically breaks platelets. Detergent lysis and room temperature methods using citrated whole blood with centrifugation are also effective [15]. Future developments in HPL production aim to improve consistency and safety through the use of standardized production protocols and larger donor pools [16]. These approaches help prevent clotting and ensure a more stable culture environment, with each method tailored to optimize growth factor release while maintaining product safety and quality.

In our previous study [11], we demonstrated the efficacy of the calcium chloride method for assessing FD-HPL quality, highlighting its sensitivity in detecting calcium ion variations and its impact on growth factors like PDGF, VEGF, and TGF-β. While effective, the Ca-method is labor-intensive and less suited for large-scale applications due to variability across different laboratories. Hence, in this study, we hypothesized that a mechanical procedure, with advantages in automation, speed, and consistency, could offer a scalable alternative to the Ca-method. We compared both methods, focusing on protein yield, stability, and bioactivity, to help users select the most appropriate approach based on operational and scalability requirements. Specifically, we aimed to determine whether the mechanical method could provide a more scalable, cost-effective alternative while maintaining or enhancing the quality of FD-HPL compared to the traditional chemical method in this current study. Additionally, we sought to assess whether the mechanical approach could provide more consistent results in terms of growth factor concentrations and the overall functional performance of MSCs, with the potential for improved clinical applicability.

## 2. Materials and Methods

### 2.1. Preparation of HPL

A total of 6 expired human platelet concentrates were collected from the Blood Bank Unit, Hospital Canselor Tuanku Muhriz, Kuala Lumpur, Malaysia (Ethical approval: JEP-2023-033). These concentrates were sourced from multiple donors to ensure sufficient volume for processing. The frozen platelet concentrates were thawed in a 37 °C water bath, and the freeze–thaw cycle was repeated. Subsequently, the pooled concentrates were centrifuged at 5000 rpm for 15 min at 4 °C. The supernatant, constituting the human platelet lysate (HPL), was collected, and the pellet was discarded. Multiple batches of platelet concentrates were pooled together to create a homogeneous preparation. This pooling was done to reduce individual donor variability and ensure consistency in the final FD-HPL product. The HPL was then divided into two portions for further processing.

### 2.2. Preparation of FD-HPL Through Calcium Salt Method

To one portion of the HPL, 20 mM of calcium chloride (CaCl_2_; Sigma-Aldrich, St. Louis, MO, USA) was added, followed by incubation at 37 °C for 2 h and then overnight at 4 °C. The clotted HPL was centrifuged at 5000 rpm for 15 min at 4 °C, and the resulting supernatant was collected and filtered through a sterile 0.22 µm filter to obtain FD-HPL.

### 2.3. Preparation of FD-HPL Through Mechanical Method

The second portion of HPL was mixed with Dulbecco’s Modified Eagle Medium (DMEM; Sigma-Aldrich, St. Louis, MO, USA) and transferred to 50 mL conical tubes. The tubes were incubated at room temperature for 4 h, followed by overnight incubation at 4 °C. Afterward, the coagulated medium was heated to 37 °C for 1 h. The tubes were then vigorously shaken and centrifuged at 5000 rpm for 10 min at room temperature. The supernatant was collected and filtered through a 0.22 µm filter to obtain FD-HPL.

### 2.4. Measurement of Fibrinogen

An enzyme-linked immunosorbent assay (ELISA) was conducted to measure fibrinogen concentrations in FD-HPL samples, following Cusabio’s protocol (Cusabio, Wuhan, China). FD-HPL samples were diluted and added to antibody-coated wells, and incubated for 1–2 h, followed by sequential additions of a detection antibody and HRP-avidin with further incubation. After adding the TMB substrate solution and incubating at 37 °C for 10–15 min in the dark, a stop solution was applied. Absorbance was read at 450 nm within 5 min using a spectrophotometric plate reader to quantify fibrinogen levels in each sample.

### 2.5. Measurement of Growth Factors

The concentrations of growth factors, specifically BDNF and VEGF, in FD-HPL prepared by the mechanical and calcium chloride methods, were measured using Biolegend ELISA kits (Biolegend, San Diego, CA, USA). FD-HPL samples were diluted with the provided diluent and added to antibody-coated wells, followed by a 2 h incubation. Detection antibodies and HRP-avidin were then added sequentially, with 1 h and 30 min incubation periods, respectively. Afterward, TMB substrate solution was added to each well and incubated for 10 min at room temperature in the dark. The reaction was stopped with a stop solution, and absorbance was measured at 450 nm using a spectrophotometric multi-well plate reader.

Meanwhile, IGF and KGF levels were measured using ELK Biotechnology ELISA kits (ELK Biotechnology, Wuhan, China). A 100 μL volume of either standard working solution or sample was added to each plate well and incubated at 37 °C for 80 min. After washing, the biotinylated antibody solution was added and incubated for 50 min, followed by incubation with streptavidin-HRP solution for another 50 min. TMB substrate solution was then added and incubated at 37 °C for 20 min. The reaction was stopped with a stop reagent, and absorbance was immediately measured at 450 nm using a microplate reader after ensuring the plate was free of bubbles or contaminants. Each experiment was performed in triplicate, and data are presented as mean ± standard error mean (SEM).

### 2.6. Culture of UC-MSCs

UC-MSCs were obtained from the Department of Tissue Engineering and Regenerative Medicine (DTERM) Primary Cell Bank. FD-HPL prepared by both the calcium chloride and mechanical methods was added to low-glucose DMEM at a 10% concentration, supplemented with a 1% antibiotic-antimycotic solution (Gibco, New York, NY, USA). The revived cells were cultured in these media and incubated at 37 °C with 5% CO_2_. The medium was changed every 2–3 days. Cells were examined under 40× magnification using an inverted microscope, and their morphology was captured daily for five days.

### 2.7. MSC Characterization

To characterize the surface markers of the cell population, flow cytometry was performed using a BD Biosciences flow cytometer (BD Biosciences, Franklin Lakes, NJ, USA). UC-MSC cell cultures were harvested, washed with phosphate-buffered saline (PBS), and then resuspended in PBS containing 2% fetal bovine serum (FBS) to block non-specific binding. Approximately 1 × 10^5^ cells were incubated with the following antibodies: anti-CD73, anti-CD90, anti-CD105, anti-CD34, and anti-CD3. Cells were incubated with the antibodies at 4 °C for 30 min in the dark. After incubation, the cells were washed twice with PBS containing 2% FBS and resuspended in PBS for analysis. Fluorescence data were collected using the flow cytometer, and the expression of CD markers was analyzed with BD FACSDiva software v. 6.1.3 (BD Biosciences, Franklin Lakes, NJ, USA). All the flow cytometry antibodies used were procured from BD Biosciences, USA. Each experiment was performed in triplicate, and data are presented as mean ± SEM.

### 2.8. Cell Morphology, Viability, and Proliferation

UC-MSCs cultured in FD-HPL prepared by both the calcium chloride and mechanical methods were observed every 3 days for morphological changes, growth patterns, and confluency using an inverted light microscope. Once the cells reached 70–80% confluence, they were trypsinized using 0.05% trypsin-EDTA (Sigma-Aldrich, St. Louis, MO, USA). The cells were then stained with trypan blue solution and counted using a hemocytometer. Results from cells cultured in FD-HPL prepared by both methods were compared. Cell viability was calculated using the formula:

Viability = (Total live cells/(Total live + dead cells)) × 100%

The population doubling time (PDT) was calculated using the following formula:

PDT = t log2/(logN2 − logN1)

where t denotes time in culture (hours); N2 denotes the cell number at the end of the passage; and N1 denotes the cell number seeded at the beginning of the passage. Each experiment was performed in triplicate, and data are presented as mean ± SEM.

### 2.9. Cell Survival Rate

The cell survival rate in FD-HPL prepared by calcium chloride and mechanical methods was determined using the CCK-8 assay (Elabscience, Wuhan, China). UC-MSCs were suspended in a culture medium supplemented with FBS to create cell suspensions, and 100 µL of the suspension was added to each well of a 96-well plate. After incubating the cells at 37 °C with 5% CO_2_ for 24 h, the culture medium containing FBS was aspirated from all wells, except for the negative control. Then, 100 µL of FD-HPL, prepared using both methods, was added to the respective wells, replacing the FBS-containing medium. The plate was incubated at 37 °C with 5% CO_2_ for 5 days, after which 10 µL of CCK-8 buffer was added to each well. Following a 4 h incubation at 37 °C in the dark, absorbance was measured at 450 nm. The average optical density (OD) values were calculated and the cell survival rate was determined using the formula:

Cell survival rate (%) = (OD of test cells/OD of negative control cells) × 100%

Each experiment was performed in triplicate, and data are presented as mean ± SEM.

### 2.10. Quantitative Gene Expression Analysis by Real-Time PCR

Real-time PCR was used to analyze the expression levels of indoleamine 2,3-dioxygenase (IDO), prostaglandin H synthase-2 (PGHS-2/COX-2), transforming growth factor beta (TGF-β), and glyceraldehyde 3-phosphate dehydrogenase (GAPDH) in the UC-MSCs treated with respective FD-HPLs. Primers were designed using Primer 3 software v. 0.4.0 and the GeneBank database. Real-time PCR reactions were performed with 100 ng of total RNA, 400 nM of each primer, and the iScript One-Step RT-PCR kit with SYBR Green (Bio-Rad, Hercules, CA, USA) based on the manufacturer’s instructions. Reactions were run on a Bio-Rad iCycler (Bio-Rad, Hercules, CA, USA) with the following profile: cDNA synthesis at 50 °C for 30 min; pre-denaturation at 94 °C for 2 min; and PCR amplification for 38 cycles (30 s at 94 °C, 30 s at 60 °C, and 30 s at 72 °C). A melt curve analysis followed to confirm reaction specificity. Gene expression was normalized to the housekeeping gene, GAPDH. Each experiment was performed in triplicate, and data are presented as mean ± SEM.

### 2.11. Statistical Analysis

Statistical analysis was performed using GraphPad Prism v.10.3.0 (GraphPad Software, Inc., San Diego, CA, USA). A two-way ANOVA with Tukey’s multiple comparison test was used for comparisons involving three or more groups, while an independent samples *t*-test was applied for comparisons between two groups. Each experiment was performed in triplicate, and data are presented as mean ± SEM, and a *p*-value of <0.05 was considered statistically significant, with ** *p* < 0.01 and *** *p* < 0.001 indicating higher levels of significance.

## 3. Results

### 3.1. Concentration of Fibrinogen

**Figure 1:**
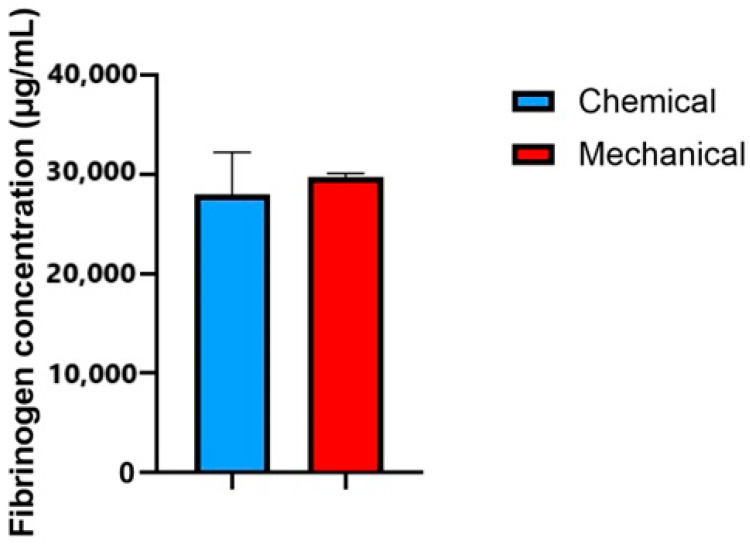
Concentration of fibrinogen measured from FD-HPL prepared using chemical and mechanical methods. Both methods yielded similar fibrinogen levels. Values are expressed as the mean ± SEM (n = 3).

The fibrinogen concentrations obtained from the two preparation methods—chemical and mechanical—were comparable, with values around 30,000 µg/mL. This similarity indicates that both methods achieve a similar level of fibrinogen depletion, with only minor variability between them (Figure 1). Although the residual fibrinogen concentration remains high in both methods, the achieved depletion appears sufficient to support UC-MSC culture without excessive fibrin interference.

### 3.2. Concentration of Growth Factors

**Figure 2:**
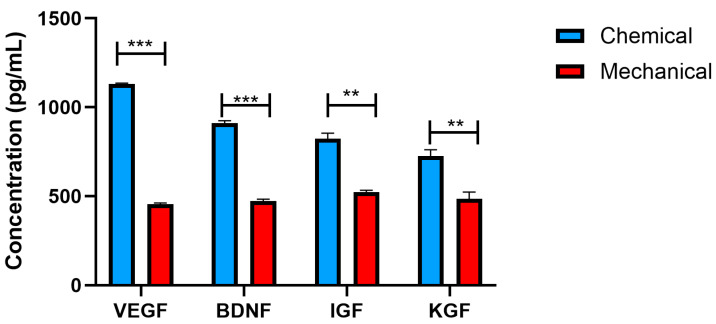
Concentrations of VEGF, BDNF, IGF, and KGF in FD-HPL prepared using calcium salt and mechanical methods. The bar graphs illustrate significant differences in growth factor concentrations between the two preparation methods. Values are expressed as the mean ± SEM (n = 3). Statistical significance is indicated as ** *p* < 0.01 and *** *p* < 0.001.

The analysis revealed a significant difference in the concentrations of VEGF, BDNF, IGF, and KGF between FD-HPL prepared using the calcium salt (chemical) method and those prepared via the mechanical method. Specifically, the concentrations were as follows: calcium salt method (VEGF: 1108.10 ± 19.131 pg/mL, BDNF: 975.12 ± 31.57 pg/mL, IGF: 957.23 ± 58.57 pg/mL, KGF: 937.26 ± 58.57 pg/mL) and mechanical method (VEGF: 487.25 ± 20.81 pg/mL, BDNF: 478.36 ± 34.58 pg/mL, IGF: 506.27 ± 58.87 pg/mL, KGF: 497.78 ± 104.57 pg/mL). The FD-HPL obtained through the calcium salt method demonstrated significantly enhanced levels of these growth factors compared to the mechanically processed FD-HPL (Figure 2). This variation suggests that the preparation technique notably influences the release and concentration of biologically active factors in FD-HPL, which may have implications for their use in regenerative medicine and therapeutic applications.

### 3.3. Morphology of UC-MSCs

**Figure 3:**
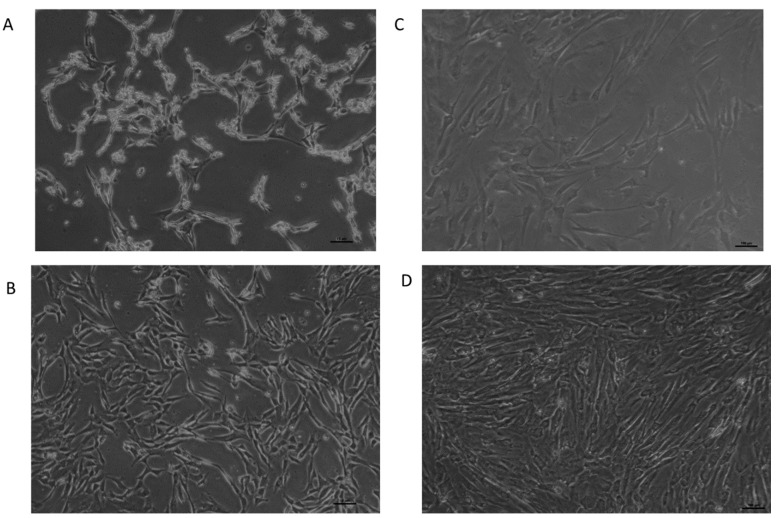
The morphology of UC-MSCs after exposure to FD-HPL prepared using chemical and mechanical methods. Panel (**A**) (Day 1): UC-MSCs exposed to mechanically prepared HPL show an initial sparse state with branched, elongated cells and large empty spaces, typical of freshly seeded cultures. Panel (**B**) (Day 5): UC-MSCs exposed to mechanically prepared HPL demonstrate increased cell density and proliferation, forming a more interconnected network while maintaining their elongated shape. Panel (**C**) (Day 1): UC-MSCs exposed to chemically prepared HPL exhibit a more fibroblastic morphology. Panel (**D**) (Day 5): UC-MSCs exposed to chemically prepared HPL display high cell density, indicating a fully confluent state with tightly packed, interconnected cells. Cells were viewed under 40× magnification.

The UC-MSCs cultured with FD-HPL prepared using both the calcium salt and mechanical methods reached approximately 80–90% confluence within five days. While both methods showed similar cell morphologies by Day 5, the FD-HPL prepared using calcium chloride resulted in superior overall outcomes, with spindle-shaped fibroblastic morphologies being much clearer, indicating that growth factors promote cell spreading and elongation. In contrast, the FD-HPL prepared by the mechanical method began to exhibit signs of clotting by Day 5. This observation demonstrates that types of FD-HPL influence the morphology of UC-MSCs (Figure 3).

### 3.4. Characterisation of UC-MSCs

**Figure 4:**
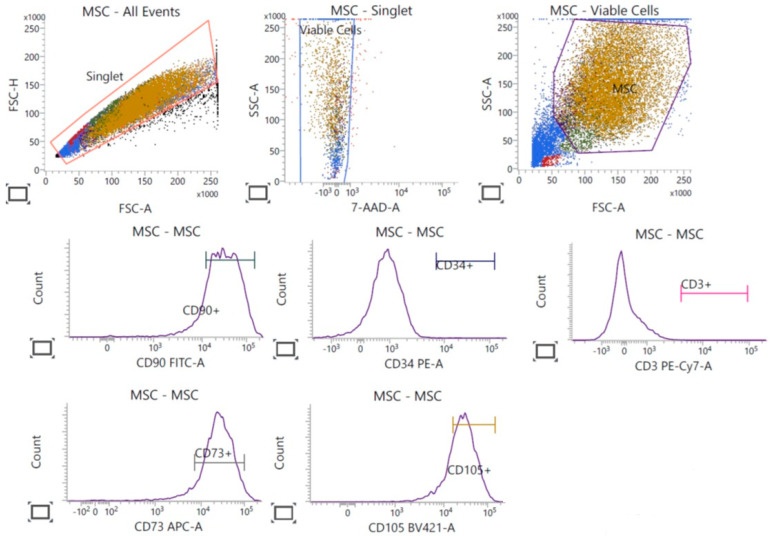
Flow cytometry analysis of UC-MSC surface markers. The UC-MSC population shows positive expression for MSC-specific markers CD73, CD90, and CD105, confirming the MSC identity of the cultured cells. Negative expression of CD34 and CD3 indicates the absence of hematopoietic and lymphoid cells, supporting the purity of the UC-MSC culture.

The characterization of UC-MSCs was performed using flow cytometry to analyze specific surface markers. The analysis confirmed that the UC-MSC population cultured with both types of HPL was positive for CD73, CD90, and CD105, which are standard markers for identifying MSCs. Additionally, the cells were negative for CD34 and CD3, where CD34 is typically associated with hematopoietic stem cells, and CD3 is a marker for T-cells (Figure 4). The absence of CD34 and CD3 in the UC-MSC culture indicates minimal contamination from hematopoietic and lymphoid cells, supporting the purity of the MSC population. This marker profile aligns well with the expected characteristics of MSCs, confirming the suitability of these cells for research or therapeutic applications involving stem cells. By ensuring the cell culture contained a pure MSC population, we can confidently attribute the observed cellular responses to the FD-HPL treatments rather than variability from non-MSC cell types. This characterization supports the validity of the study’s findings and ensures that the effects of FD-HPL on UC-MSCs are accurately interpreted.

### 3.5. Morphological Properties of Cells

**Figure 5:**
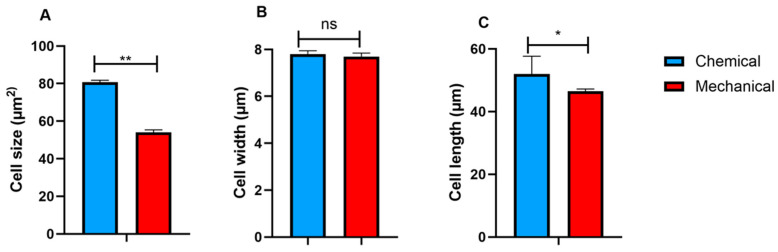
Analysis of UC-MSC cell size cultured in various FD-HPL supplements revealed a significant difference in cell size between the two groups (**A**). While the widths of UC-MSCs under the same conditions were assessed, no significant differences were observed (**B**). In contrast, the lengths of UC-MSCs cultured in different FD-HPL supplements showed a significant difference, with mechanically prepared FD-HPL exhibiting a decrease in cell length compared to chemically prepared FD-HPL (**C**). Values are presented as mean ± SEM (n = 3). Statistical significance is indicated as * *p* < 0.05 and ** *p* < 0.01. ns: not significant.

The UC-MSCs cultured with FD-HPL prepared using the chemical method exhibited larger cell size compared to those cultured with FD-HPL prepared by the mechanical method, and this difference was statistically significant (Figure 5A). The enhanced cell size suggests that the chemical preparation method may create a more favorable environment for cellular growth and expansion. However, no significant differences in cell width were observed between the two groups, indicating that while the chemical method promotes overall cell size and length, it does not markedly affect cell width (Figure 5B). Additionally, cell length was greater in cells cultured with chemically prepared FD-HPL, further supporting the notion that this method positively influences cell morphology (Figure 5C). This study underscores the importance of the FD-HPL preparation method in influencing the morphological characteristics of UC-MSCs.

### 3.6. The Cell Viability, Yield, and Proliferation

**Figure 6:**
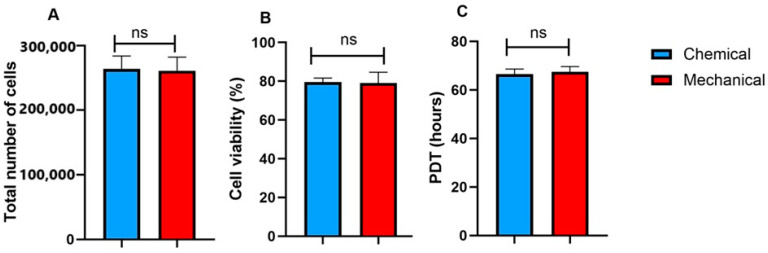
There were no statistically significant differences between chemical and mechanical treatments across all measured parameters, suggesting comparable efficacy of both methods in maintaining cell number (**A**), viability (**B**), and growth rate (**C**). Data are presented as mean ± SEM (n = 3). ns: not significant.

There was no significant difference in the total cell number between UC-MSCs cultured with chemically prepared FD-HPL (256,000 ± 17,500 cells) and those cultured with mechanically prepared FD-HPL (248,800 ± 19,600 cells), indicating that both preparation methods yield comparable cell proliferation rates (Figure 6A). Similarly, cell viability remained consistent across both groups, with chemically prepared FD-HPL showing 78.5 ± 9.50% viability and mechanically prepared FD-HPL showing 78.2 ± 16.50% (Figure 6B). This further demonstrates that the preparation method did not significantly impact the health or survival of the cells. Additionally, the population doubling time (PDT), which reflects the time it takes for the cell population to double, was measured for both groups. The PDT for cells cultured with chemically prepared FD-HPL was approximately 67 ± 9.45 h, while the PDT for the mechanically prepared FD-HPL group was 69 ± 10.31 h (Figure 6C). Again, no significant difference was observed between the two treatments, suggesting that both methods support similar rates of cell division and growth.

### 3.7. Cell Survival Rate

**Figure 7:**
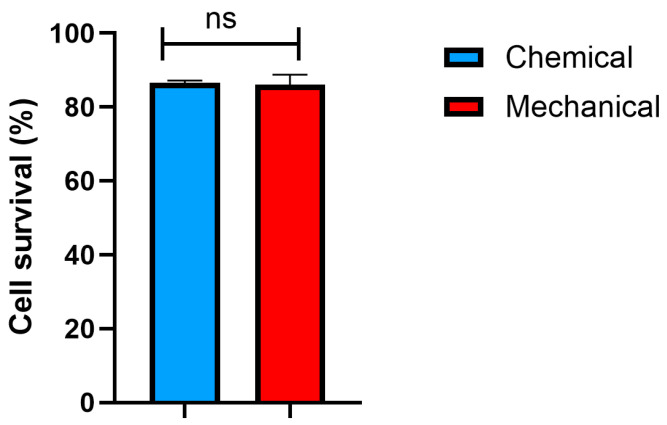
The cell survival rates of UC-MSCs were similar for both preparation methods. This indicates high cell viability and comparable efficacy in maintaining viable cell populations. Data are presented as mean ± SEM (n = 3). ns: not significant.

The mechanical treatment demonstrated a slightly higher cell survival rate, with approximately 88% of cells exhibiting metabolic activity compared to 86% for cells cultured with chemically prepared FD-HPL (Figure 7). Despite this minor difference, both preparation methods yielded similarly high cell survival rates, indicating that the overall viability of UC-MSCs was not significantly affected by the method of FD-HPL preparation.

### 3.8. Expression of Immunomodulatory Genes

**Figure 8:**
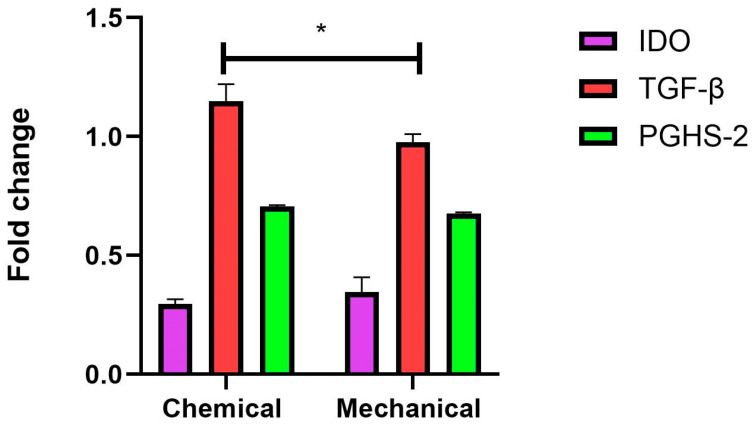
Fold change in expression of IDO, TGF-β, and PGHS-2 under chemical and mechanical methods. The findings demonstrate the relative expression levels of three markers across two experimental conditions. Under chemical conditions, TGF-β shows the highest expression, followed by PGHS-2 and IDO with the lowest expression. Under mechanical conditions, TGF-β remains the most expressed marker, although slightly lower, with PGHS-2 and IDO showing similar trends. Values are presented as mean ± SEM (n = 3). Statistical significance is indicated as * *p* < 0.05.

The expression levels of immunomodulatory genes, including IDO, TGF-β, and PGHS-2, were assessed under two preparations, chemical and mechanical, to evaluate differential responses. Under chemical conditions, TGF-β showed the highest expression level, with an approximately 1.1-fold increase, followed by PGHS-2 at around 0.7-fold, and IDO at 0.3-fold. In contrast, under mechanical conditions, TGF-β expression was slightly reduced to approximately 0.95-fold, while PGHS-2 and IDO showed marginal changes, with values around 0.65-fold and 0.35-fold, respectively. Statistical analysis indicated a significant difference in marker expression between the chemical and mechanical methods, particularly for TGF-β (*p* < 0.05) (Figure 8). This indicates that the chemical condition generally promotes stronger expression of these markers compared to the mechanical condition, with TGF-β showing the most pronounced response.

## 4. Discussion

The use of HPL as a serum supplement for MSC culture has garnered significant interest in regenerative medicine due to its rich growth factor content that supports cell proliferation and viability [17]. Research indicates that higher growth factor concentrations can enhance MSC proliferation and migration. For instance, Li et al. (2021) demonstrated that concentrated growth factors significantly improved the proliferation and migration of dental pulp stem cells (DPSCs), underscoring the importance of the preparation method [18]. Similarly, Kazemnejad et al. (2008) reported that human bone marrow-derived MSCs cultured with HPL exhibited a threefold increase in proliferation rates compared to those in FBS [19]. Aldahmash et al. (2011) also found that human serum sources, such as platelet lysate, effectively promoted the growth of human multipotent stromal cells, highlighting the potential of human-derived alternatives to FBS [20].

FD-HPL stands out due to its enhanced growth factor profile, which promotes cell proliferation and viability, addresses clotting issues, and is relevant to human applications [11,21]. Additionally, its versatility, reproducibility, and favorable safety profile make it a valuable option. FD-HPL can be prepared using several methods, each with distinct advantages. The calcium chloride method activates platelets to form clots and removes the fibrinogen-rich supernatant; it is cost-effective and rapid but may lead to variability in growth factor concentrations [14,22]. The mechanical method involves physically disrupting platelets, offering gentle processing with lower contamination risk but requiring specialized equipment [23,24]. The freeze–thaw method is simple but may degrade sensitive proteins. Enzymatic digestion with collagenase allows for the targeted release of specific growth factors but needs careful optimization to avoid over-digestion [25]. Ultimately, the choice of preparation method for FD-HPL depends on growth factor preservation, ease of use, and the intended application in cell culture or regenerative medicine [26]. In this study, we compared two FD-HPL preparation methods whereby calcium chloride-induced fibrinogen depletion and the mechanical method to assess their impact on growth factor preservation and the functional characteristics of UC-MSCs.

The significant variation in growth factor concentrations underscores the crucial role of preparation techniques in influencing the bioactive potential of FD-HPL. Elevated levels of VEGF, BDNF, IGF, and KGF in calcium chloride-prepared FD-HPL suggest that this method may be more effective in preserving and enhancing these factors, which are essential for various regenerative processes [27]. The calcium salt method boosts growth factor concentrations beneficial for UC-MSC proliferation. Key factors like VEGF, BDNF, IGF, and KGF play vital roles in enhancing the therapeutic potential of UC-MSCs. VEGF supports angiogenesis, creating a favorable environment for UC-MSCs in tissue engineering [28]. BDNF contributes to neuroprotection and neuronal growth, making it valuable for targeting neurodegenerative conditions [29]. IGF enhances cell proliferation and inhibits apoptosis, improving UC-MSC survival and expansion [30]. KGF promotes keratinocyte proliferation, aiding wound healing and skin regeneration [31]. In contrast, the mechanical method, while beneficial for gentle processing, results in lower concentrations of these growth factors, which may limit its effectiveness in applications requiring high growth factor availability [32]. These findings align with earlier studies highlighting how preparation methods influence the release and concentration of biologically active factors in platelet lysates, affecting their therapeutic efficacy [27,28,29,30,31]. However, in our study, both methods demonstrated high fibrinogen retention, initially seeming suboptimal for cell culture. Meanwhile, positive outcomes revealed that partial fibrinogen depletion created a suitable environment for UC-MSC culture [33]. This balance reduces clotting risk while maintaining essential growth factors, enhancing UC-MSC proliferation and function for therapeutic or research applications [34]. Optimizing the depletion protocol could achieve lower levels, improving reproducibility in FD-HPL batches.

In this study, flow cytometry analysis was used to confirm the identity and purity of the UC-MSC population by examining specific surface markers. The UC-MSCs showed strong positive expression for CD73, CD90, and CD105, which are recognized markers for MSCs and are linked to key MSC properties, such as adhesion, migration, and differentiation potential [35]. These markers affirm the functional characteristics of the UC-MSCs and suggest their readiness for applications that rely on these properties. Additionally, the cells were negative for CD34 and CD3, markers for hematopoietic stem cells and T-cells, respectively. The lack of CD34 and CD3 expression indicates minimal contamination from hematopoietic or lymphoid cells, thereby confirming the purity of the MSC culture [36]. This specific marker profile aligns well with established MSC characteristics, underscoring the suitability of these UC-MSCs for both research and therapeutic applications where high purity and functional integrity of MSCs are critical. Since the MSC identity was confirmed, we used this population to assess the functional properties of FD-HPL prepared through chemical and mechanical methods. UC-MSCs cultured with calcium salt-prepared FD-HPL exhibited more pronounced spindle-shaped fibroblastic morphologies, indicating enhanced cell spreading and elongation. The spindle-shaped morphology, often associated with greater cell motility and interaction with the extracellular matrix, is vital for MSCs involved in tissue repair and regeneration [37]. This morphological difference may reflect the superior growth factor profile in calcium chloride-prepared FD-HPL, including higher concentrations of IGF and KGF, which stimulate cell proliferation, survival, and differentiation [17,38]. In contrast, clotting observed in UC-MSCs cultured with mechanically prepared FD-HPL by Day 5 raises concerns about the stability and bioactivity of the growth factors, potentially hindering cell interaction with these factors and limiting the therapeutic potential of UC-MSCs.

The chemical preparation method offers a more favorable environment for cell growth and expansion, likely due to higher concentrations of key growth factors like IGF and BDNF, which promote cell proliferation and elongation. The larger cell sizes observed in the chemically prepared FD-HPL group are likely due to increased growth factor concentrations that promote cellular metabolism and proliferation. The greater cell length in UC-MSCs cultured with FD-HPL may indicate enhanced cytoskeletal organization and cellular dynamics, likely attributed to higher levels of BDNF and VEGF [39]. The stability of cell width between groups suggests that the chemical method primarily promotes longitudinal cellular expansion rather than lateral growth, which is crucial for tissue engineering applications [40]. These findings highlight the importance of FD-HPL preparation techniques in optimizing UC-MSC characteristics for effective use in tissue engineering and regenerative medicine.

Both methods were equally effective in supporting cell growth and proliferation, as indicated by similar total cell counts and consistent viability rates. While the mechanical method may result in slightly faster cell division, both methods sustain cell health and proliferation without significant differences. In addition, IDO, TGF-β, and PGHS-2 expression in UC-MSCs varied under chemical and mechanical preparation conditions, influencing their bioactive profile. The chemical method showed higher TGF-β expression, suggesting better support for immunomodulation and regeneration [41]. In contrast, the mechanical method had lower expression levels, potentially impacting MSC functionality in regenerative applications. The chemical method may be more effective in promoting factors related to MSC proliferation and immune regulation. Overall, this suggests that both methods are suitable for research and clinical use, offering options to enhance the therapeutic potential of UC-MSCs in regenerative medicine [11,42]. Future research should aim to optimize preparation methods for improved outcomes in specific applications, advancing the efficacy of stem cell therapies using FD-HPL.

Despite the promising findings of this study, several limitations warrant consideration. First, the research primarily focused on two preparation methods for FD-HPL through calcium chloride and mechanical methods, limiting the assessment of other potential techniques that might yield different results. Additionally, the study evaluated the effects of FD-HPL on UC-MSCs in a controlled laboratory setting, which may not fully replicate the complexities of in vivo environments where multiple biological factors interact. Furthermore, the long-term effects of the different FD-HPL preparations on UC-MSC behavior, differentiation, and functional outcomes were not explored, necessitating further investigation.

## 5. Conclusions

This study demonstrates that FD-HPL prepared by both chemical and mechanical methods effectively supports UC-MSC proliferation, viability, and functionality, highlighting FD-HPL as a viable serum supplement in regenerative medicine. The chemical preparation method showed a notable advantage in promoting growth factor concentrations, such as TGF-β, IGF, and BDNF, which enhance cell proliferation, elongation, and immunomodulatory properties that are crucial for tissue engineering and therapeutic applications. The mechanical method, while yielding slightly lower growth factor levels, still provided a supportive environment for UC-MSC culture, suggesting it may be suitable in applications where gentler platelet processing is preferred. Both methods resulted in high fibrinogen retention, yet partial depletion appeared sufficient to foster a culture environment beneficial for UC-MSCs, balancing clotting risk with growth factor availability. However, the morphological differences observed, including increased spindle-shaped elongation in the chemically prepared FD-HPL group, suggest this method may be more suitable when enhanced cellular interaction and motility are desired. Overall, the findings underscore the importance of selecting FD-HPL preparation methods that align with specific therapeutic goals. Future optimization of FD-HPL preparation could further enhance consistency and effectiveness, advancing the potential of UC-MSC-based therapies in regenerative medicine.

## Data Availability

Data will be available upon request.

## References

[1] Lee D.Y., Lee S.Y., Yun S.H., Jeong J.W., Kim J.H., Kim H.W., Choi J.S., Kim G.D., Joo S.T., Choi I. (2022). Review of the Current Research on Fetal Bovine Serum and the Development of Cultured Meat. Food Sci. Anim. Resour..

[2] Liu S., Yang W., Li Y., Sun C. (2023). Fetal bovine serum, an important factor affecting the reproducibility of cell experiments. Sci. Rep..

[3] Lee D.Y., Yun S.H., Lee S.Y., Lee J., Mariano E., Joo S.T., Choi I., Choi J.S., Kim G.D., Lee J. (2023). Analysis of commercial fetal bovine serum (FBS) and its substitutes in the development of cultured meat. Food Res. Int..

[4] Pilgrim C.R., McCahill K.A., Rops J.G., Dufour J.M., Russell K.A., Koch T.G. (2022). A Review of Fetal Bovine Serum in the Culture of Mesenchymal Stromal Cells and Potential Alternatives for Veterinary Medicine. Front. Vet. Sci..

[5] Subbiahanadar Chelladurai K., Selvan Christyraj J.D., Rajagopalan K., Yesudhason B.V., Venkatachalam S., Mohan M., Chellathurai Vasantha N., Selvan Christyraj J.R.S. (2021). Alternative to FBS in animal cell culture—An overview and future perspective. Heliyon.

[6] Piletz J.E., Drivon J., Eisenga J., Buck W., Yen S., McLin M., Meruvia W., Amaral C., Brue K. (2018). Human Cells Grown with or without Substitutes for Fetal Bovine Serum. Cell Med..

[7] Guiotto M., Raffoul W., Hart A.M., Riehle M.O., di Summa P.G. (2020). Human platelet lysate to substitute fetal bovine serum in hMSC expansion for translational applications: A systematic review. J. Transl. Med..

[8] Seidelmann N., Duarte Campos D.F., Rohde M., Johnen S., Salla S., Yam G.H., Mehta J.S., Walter P., Fuest M. (2021). Human platelet lysate as a replacement for fetal bovine serum in human corneal stromal keratocyte and fibroblast culture. J. Cell. Mol. Med..

[9] Oeller M., Laner-Plamberger S., Krisch L., Rohde E., Strunk D., Schallmoser K. (2021). Human Platelet Lysate for Good Manufacturing Practice-Compliant Cell Production. Int. J. Mol. Sci..

[10] Palombella S., Perucca Orfei C., Castellini G., Gianola S., Lopa S., Mastrogiacomo M., Moretti M., de Girolamo L. (2022). Systematic review and meta-analysis on the use of human platelet lysate for mesenchymal stem cell cultures: Comparison with fetal bovine serum and considerations on the production protocol. Stem Cell Res. Ther..

[11] Kee L.T., Lee Y.T., Ng C.Y., Hassan M.N.F., Ng M.H., Mahmood Z., Abdul Aziz S., Law J.X. (2023). Preparation of Fibrinogen-Depleted Human Platelet Lysate to Support Heparin-Free Expansion of Umbilical Cord-Derived Mesenchymal Stem Cells. Biology.

[12] Mareschi K., Castiglia S., Adamini A., Rustichelli D., Marini E., Banche Niclot A.G.S., Bergallo M., Labanca L., Ferrero I., Fagioli F. (2020). Inactivated Platelet Lysate Supports the Proliferation and Immunomodulant Characteristics of Mesenchymal Stromal Cells in GMP Culture Conditions. Biomedicines.

[13] Kandoi S., Kumar P., Patra B., Vidyasekar P., Sivanesan D., Verma R.S. (2018). Evaluation of platelet lysate as a substitute for FBS in explant and enzymatic isolation methods of human umbilical cord MSCs. Sci. Rep..

[14] Mouloud Y., Staubach S., Stambouli O., Mokhtari S., Kutzner T.J., Zwanziger D., Hemeda H., Giebel B. (2024). Calcium chloride declotted human platelet lysate promotes the expansion of mesenchymal stromal cells and allows manufacturing of immunomodulatory active extracellular vesicle products. Cytotherapy.

[15] Naskou M.C., Sumner S., Berezny A., Copland I.B., Peroni J.F. (2019). Fibrinogen-Depleted Equine Platelet Lysate Affects the Characteristics and Functionality of Mesenchymal Stem Cells. Stem Cells Dev..

[16] Naskou M.C., Sumner S.M., Chocallo A., Kemelmakher H., Thoresen M., Copland I., Galipeau J., Peroni J.F. (2018). Platelet lysate as a novel serum-free media supplement for the culture of equine bone marrow-derived mesenchymal stem cells. Stem Cell Res. Ther..

[17] Cañas-Arboleda M., Beltrán K., Medina C., Camacho B., Salguero G. (2020). Human Platelet Lysate Supports Efficient Expansion and Stability of Wharton’s Jelly Mesenchymal Stromal Cells via Active Uptake and Release of Soluble Regenerative Factors. Int. J. Mol. Sci..

[18] Li Z., Liu L., Wang L., Song D. (2021). The effects and potential applications of concentrated growth factor in dentin-pulp complex regeneration. Stem Cell Res. Ther..

[19] Kazemnejad S., Allameh A., Gharehbaghian A., Soleimani M., Amirizadeh N., Jazayeri M. (2008). Efficient replacing of fetal bovine serum with human platelet releasate during propagation and differentiation of human bone-marrow-derived mesenchymal stem cells to functional hepatocyte-like cells. Vox Sang..

[20] Aldahmash A., Haack-Sørensen M., Al-Nbaheen M., Harkness L., Abdallah B.M., Kassem M. (2011). Human serum is as efficient as fetal bovine serum in supporting proliferation and differentiation of human multipotent stromal (mesenchymal) stem cells in vitro and in vivo. Stem Cell Rev. Rep..

[21] Laner-Plamberger S., Lener T., Schmid D., Streif D.A., Salzer T., Öller M., Hauser-Kronberger C., Fischer T., Jacobs V.R., Schallmoser K. (2015). Mechanical fibrinogen-depletion supports heparin-free mesenchymal stem cell propagation in human platelet lysate. J. Transl. Med..

[22] Yeh W.T., Yu E.Y., Lu Y.H., Livkisa D., Burnouf T., Lundy D.J. (2024). Bioprocessing of human platelet concentrates to generate lysates and extracellular vesicles for therapeutic applications. MethodsX.

[23] Duarte Rojas J.M., Restrepo Múnera L.M., Estrada Mira S. (2024). Comparison between Platelet Lysate, Platelet Lysate Serum, and Fetal Bovine Serum as Supplements for Cell Culture, Expansion, and Cryopreservation. Biomedicines.

[24] Almeria C., Kreß S., Weber V., Egger D., Kasper C. (2022). Heterogeneity of mesenchymal stem cell-derived extracellular vesicles is highly impacted by the tissue/cell source and culture conditions. Cell Biosci..

[25] Sugar A., Hussain M., Chamberlain W., Dana R., Kelly D.P., Ta C., Irvine J., Daluvoy M., Perez V., Olson J. (2022). Cambium Dry Eye Study Group A Randomized Trial of Topical Fibrinogen-Depleted Human Platelet Lysate Treatment of Dry Eye Secondary to Chronic Graft-versus-Host Disease. Ophthalmol. Sci..

[26] Palombella S., Guiotto M., Higgins G.C., Applegate L.L., Raffoul W., Cherubino M., Hart A., Riehle M.O., di Summa P.G. (2020). Human platelet lysate as a potential clinical-translatable supplement to support the neurotrophic properties of human adipose-derived stem cells. Stem Cell Res. Ther..

[27] Yan L., Zhou L., Yan B., Zhang L., Du W., Liu F., Yuan Q., Tong P., Shan L., Efferth T. (2020). Growth factors-based beneficial effects of platelet lysate on umbilical cord-derived stem cells and their synergistic use in osteoarthritis treatment. Cell Death Dis..

[28] Mastrullo V., Cathery W., Velliou E., Madeddu P., Campagnolo P. (2020). Angiogenesis in Tissue Engineering: As Nature Intended?. Front. Bioeng. Biotechnol..

[29] Azman K.F., Zakaria R. (2022). Recent Advances on the Role of Brain-Derived Neurotrophic Factor (BDNF) in Neurodegenerative Diseases. Int. J. Mol. Sci..

[30] Youssef A., Aboalola D., Han V.K. (2017). The Roles of Insulin-Like Growth Factors in Mesenchymal Stem Cell Niche. Stem Cells Int..

[31] Pierce G.F., Yanagihara D., Klopchin K., Danilenko D.M., Hsu E., Kenney W.C., Morris C.F. (1994). Stimulation of all epithelial elements during skin regeneration by keratinocyte growth factor. J. Exp. Med..

[32] Burnouf T., Strunk D., Koh M.B., Schallmoser K. (2016). Human platelet lysate: Replacing fetal bovine serum as a gold standard for human cell propagation?. Biomaterials.

[33] Hoang V.T., Le D.S., Hoang D.M., Phan T.T.K., Ngo L.A.T., Nguyen T.K., Bui V.A., Nguyen Thanh L. (2024). Impact of tissue factor expression and administration routes on thrombosis development induced by mesenchymal stem/stromal cell infusions: Re-evaluating the dogma. Stem Cell Res. Ther..

[34] Fernández-Santos M.E., Garcia-Arranz M., Andreu E.J., García-Hernández A.M., López-Parra M., Villarón E., Sepúlveda P., Fernández-Avilés F., García-Olmo D., Prosper F. (2022). Optimization of Mesenchymal Stromal Cell (MSC) Manufacturing Processes for a Better Therapeutic Outcome. Front. Immunol..

[35] Lin C.S., Xin Z.C., Dai J., Lue T.F. (2013). Commonly used mesenchymal stem cell markers and tracking labels: Limitations and challenges. Histol. Histopathol..

[36] Bucar S., Branco A.D.M., Mata M.F., Milhano J.C., Caramalho Í., Cabral J.M.S., Fernandes-Platzgummer A., da Silva C.L. (2021). Influence of the mesenchymal stromal cell source on the hematopoietic supportive capacity of umbilical cord blood-derived CD34^+^-enriched cells. Stem Cell Res. Ther..

[37] Zhou T., Yuan Z., Weng J., Pei D., Du X., He C., Lai P. (2021). Challenges and advances in clinical applications of mesenchymal stromal cells. J. Hematol. Oncol..

[38] Hefka Blahnova V., Dankova J., Rampichova M., Filova E. (2020). Combinations of growth factors for human mesenchymal stem cell proliferation and osteogenic differentiation. Bone Jt. Res..

[39] Wang S., Yu L., Sun M., Mu S., Wang C., Wang D., Yao Y. (2013). The therapeutic potential of umbilical cord mesenchymal stem cells in mice premature ovarian failure. BioMed Res. Int..

[40] Ross A.M., Jiang Z., Bastmeyer M., Lahann J. (2012). Physical aspects of cell culture substrates: Topography, roughness, and elasticity. Small.

[41] Sarsenova M., Kim Y., Raziyeva K., Kazybay B., Ogay V., Saparov A. (2022). Recent advances to enhance the immunomodulatory potential of mesenchymal stem cells. Front. Immunol..

[42] Bagno L.L., Salerno A.G., Balkan W., Hare J.M. (2022). Mechanism of Action of Mesenchymal Stem Cells (MSCs): Impact of delivery method. Expert Opin. Biol. Ther..

